# Supplementary material for: Efficacy and mechanism of action of cipargamin as an antibabesial drug candidate
Source: eLife. 2025 Jun 19;13:RP101128. doi: 10.7554/eLife.101128 (PMC12178600; doi:10.7554/eLife.101128)
Supplement: Supplementary file 1. [file elife-101128-supp1.docx]

**Supplementary file 1. Primer sets of *B. gibsoni* ATP4.**

| Gene | Primer (5'-3') |
| --- | --- |
| *Bgatp4*-1F | ACGTAGTTGTCGAACATCTTACACCAATGC |
| *Bgatp4*-1R | CAGAGGTAGCATAGTGAGATCGCCCCGA |
| *Bgatp4*-2F | GAGATTGCAAGGTTTACGGATGAGTCTATGG |
| *Bgatp4*-2R | ATGACAACGTCACCGGGTACAACACGCTTG |
| *Bgatp4*-3F | GAAAAGCTTGCTCAAATGTCTTCACCGACAAC |
| *Bgatp4*-3R | GTTTCTGGACGAGTTGGATCCTCATAACC |
| *Bgatp4*-4F | AAATTGGGGGGGATAATTGGCATTATCTCC |
| *Bgatp4*-4R | ACCTTATCAAAGGGTACCTCTTGCTTTG |
| *Bgatp4*-5F | TCAATCCATTCGGTGGAATTTTCAAAAAGG |
| *Bgatp4*-5R | GTGTTTGAATAACATATCTGGTGCCCCCTT |
| *Bgatp4*-6F | GGTAAAGAACAGATTTGCTGACATAGATCT |
| *Bgatp4*-6R | ACCCTATTTCTTGTGCGATGGCCGTAGCTG |
| *Bgatp4*-7F | AGGGACGCTATAAATACTTGTGGAAAGGC |
| *Bgatp4*-7R | GTACGCCCAGACTCGATTGAGTTAACTATTG |
| *Bgatp4*-8F | GGGTATTAATGGCACAGATGTTGCAAAAGG |
| *Bgatp4*-8R | GACAGTTCCCACTAGCATCATTAAGTGTAA |
| *Bgatp4*-9F | ATCATCATTGCGCTATATGTTTCTACCGG |
| *Bgatp4*-9R | CTAATGGGGTAGCATTTCCATTCTCAACAA |
| *Bgatp4*-10F | AGGGAGTTCCTCCCATGTATAAAGATACCT |
| *Bgatp4*-10R | TTAGGCTTCTGCCCTTTTCATTTTTCGCTT |
