# Supplementary material for: Efficacy and mechanism of action of cipargamin as an antibabesial drug candidate
Source: eLife. 2025 Jun 19;13:RP101128. doi: 10.7554/eLife.101128 (PMC12178600; doi:10.7554/eLife.101128)
Supplement: Supplementary file 2. [file elife-101128-supp2.docx]

**Supplementary file 2. Primer sets of *B. microti* ATP4.**

| Gene | Primer (5'-3') |
| --- | --- |
| *Bmatp4*-1F | ATGAGTAGATCTCAGTCTAACATTTC |
| *Bmatp4*-1R | TGCTATTAGTAGTGCTATAACAAAG |
| *Bmatp4*-2F | CAAGTATGGCGAAAATGCTATAAAC |
| *Bmatp4*-2R | CATTCCGTTTGTTACGCTGGTGC |
| *Bmatp4*-3F | CTTACAGGTGAGAGTGAAGATGTAAAG |
| *Bmatp4*-3R | ACAAATTGCTGAGCAGCAACCCAGAG |
| *Bmatp4*-4F | CTATTTCATTGTCATTGGGAGCTAA |
| *Bmatp4*-4R | CAAATCCGGCCTTAGCTGCGCCAA |
| *Bmatp4*-5F | TCATACGGTTCAACGTTATCACATG |
| *Bmatp4*-5R | TCTATCCGACGACTCCAACCTTTC |
| *Bmatp4*-6F | GTTGTCCAAGAATGCTTATAGGGTG |
| *Bmatp4*-6R | CATTGCCACGCCAATGTCCGCCTGTTT |
| *Bmatp4*-7F | GTTAACTCGTTGAAGAGACAAGGTTAC |
| *Bmatp4*-7R | CACAATTGTACCGACAACTGCCGCTG |
| *Bmatp4*-8F | GGCCAAAATCTCAGCCTTTAATGAC |
| *Bmatp4*-8R | CAGCTCCAATGGGTTGGTAGTAACCATT |
| *Bmatp4*-9F | CGTTATTGACGGCGTTGAGAATGATG |
| *Bmatp4*-9R | CCCAAAACACCTTTGCCATTTAACTC |
| *Bmatp4*-10F | GAAAATTGTAGAGCATTTCAAAACCAG |
| *Bmatp4*-10R | TTATTGCAGGGCAGCCTGCTTAGA |
