# Supplementary material for: Efficacy and mechanism of action of cipargamin as an antibabesial drug candidate
Source: eLife. 2025 Jun 19;13:RP101128. doi: 10.7554/eLife.101128 (PMC12178600; doi:10.7554/eLife.101128)
Supplement: Supplementary file 3. [file elife-101128-supp3.docx]

**Supplementary file 3. Interactions of CIP from docking simulations.**

| Receptor and binding energy | Name | Distance | Category | Types |
| --- | --- | --- | --- | --- |
| BgATP4^WT^  -6.43 kcal/mol | GLY407 | 2.67648 | Hydrogen Bond; Halogen | Carbon Hydrogen Bond; Halogen (Fluorine) |
|  | CYS959 | 3.2396 | Halogen | Halogen (Cl, Br, I) |
|  | VAL962 | 2.66882 | Hydrophobic | Pi-Sigma |
|  | CYS959 | 4.38742 | Hydrophobic | Alkyl |
|  | VAL962 | 4.29249 | Hydrophobic | Alkyl |
|  | ALA963 | 3.8231 | Hydrophobic | Alkyl |
|  | ALA1202 | 3.67015 | Hydrophobic | Alkyl |
|  | MET211 | 5.06565 | Hydrophobic | Alkyl |
|  | VAL962 | 4.25668 | Hydrophobic | Alkyl |
|  | ILE414 | 4.70605 | Hydrophobic | Alkyl |
|  | CYS959 | 4.83891 | Hydrophobic | Pi-Alkyl |
|  | VAL962 | 4.98402 | Hydrophobic | Pi-Alkyl |
|  | VAL411 | 5.43239 | Hydrophobic | Pi-Alkyl |
|  | VAL962 | 5.05537 | Hydrophobic | Pi-Alkyl |
|  | PHE954 | 4.07586 | Hydrophobic | Pi-Alkyl |
| BgATP4^L921V^ -6.40 kcal/mol | GLY407 | 2.69013 | Hydrogen Bond; Halogen | Carbon Hydrogen Bond; Halogen (Fluorine) |
|  | CYS959 | 3.22066 | Halogen | Halogen (Cl, Br, I) |
|  | VAL962 | 2.66391 | Hydrophobic | Pi-Sigma |
|  | CYS959 | 4.35422 | Hydrophobic | Alkyl |
|  | VAL962 | 4.31879 | Hydrophobic | Alkyl |
|  | ALA963 | 3.8209 | Hydrophobic | Alkyl |
|  | ALA1202 | 3.63038 | Hydrophobic | Alkyl |
|  | MET211 | 5.0878 | Hydrophobic | Alkyl |
|  | VAL962 | 4.24186 | Hydrophobic | Alkyl |
|  | ILE414 | 4.71819 | Hydrophobic | Alkyl |
|  | CYS959 | 4.82036 | Hydrophobic | Pi-Alkyl |
|  | VAL962 | 4.99879 | Hydrophobic | Pi-Alkyl |
|  | VAL411 | 5.4334 | Hydrophobic | Pi-Alkyl |
|  | VAL962 | 5.04532 | Hydrophobic | Pi-Alkyl |
|  | PHE954 | 4.07482 | Hydrophobic | Pi-Alkyl |
| BgATP4^L921I^ -6.26 kcal/mol | GLY958 | 2.92346 | Hydrogen Bond; Halogen | Carbon Hydrogen Bond; Halogen (Fluorine) |
|  | GLY958 | 3.3301 | Halogen | Halogen (Fluorine) |
|  | MET410 | 5.88505 | Other | Pi-Sulfur |
|  | VAL955 | 4.31406 | Hydrophobic | Alkyl |
|  | MET410 | 3.69023 | Hydrophobic | Alkyl |
|  | VAL411 | 4.83101 | Hydrophobic | Alkyl |
|  | VAL955 | 4.79513 | Hydrophobic | Alkyl |
|  | VAL955 | 4.84729 | Hydrophobic | Alkyl |
|  | CYS959 | 4.1269 | Hydrophobic | Alkyl |
|  | ILE1205 | 4.82529 | Hydrophobic | Alkyl |
|  | CYS959 | 5.21399 | Hydrophobic | Alkyl |
|  | VAL955 | 4.90527 | Hydrophobic | Pi-Alkyl |
|  | PHE954 | 5.03012 | Hydrophobic | Pi-Alkyl |
